# Shorter Diagnostic Delay in Polish Adult Patients With Common Variable Immunodeficiency and Symptom Onset After 1999

**DOI:** 10.3389/fimmu.2020.00982

**Published:** 2020-05-27

**Authors:** Marcin Ziętkiewicz, Ewa Więsik-Szewczyk, Aleksandra Matyja-Bednarczyk, Katarzyna Napiórkowska-Baran, Zbigniew Zdrojewski, Karina Jahnz-Różyk

**Affiliations:** ^1^Department of Internal Medicine, Connective Tissue Diseases and Geriatrics, Medical University of Gdansk, Gdansk, Poland; ^2^Department of Internal Medicine, Pneumonology, Allergology and Clinical Immunology, Central Clinical Hospital of the Ministry of National Defense, Military Institute of Medicine, Warsaw, Poland; ^3^Outpatient Clinic for the Immunological and Hypercoagulable Diseases, The University Hospital in Krakow, Kraków, Poland; ^4^Department of Allergology, Clinical Immunology and Internal Diseases, Ludwik Rydygier Collegium Medicum in Bydgoszcz Nicolaus Copernicus University in Torun, Bydgoszcz, Poland

**Keywords:** primary antibody deficiency, hypogammaglobulinemia, common variable immunodeficiency, diagnostic delay, adults, epidemiology

## Abstract

Common variable immunodeficiency (CVID) is the most clinically significant primary antibody immunodeficiency recognized in adulthood. Previously published data have shown an average diagnostic delay of 10 years for Polish adult patients with CVID. In the current study, we aimed to analyze the current diagnostic delay of adult patients with CVID in Poland. To this end, we identified patients from four immunological centers specialized in the care of adult patients with primary immunodeficiencies (PID). Demographic and clinical data of patients were collected using an internet database. We identified 103 adult patients (F:M 44.7%:55.3%) in Poland with CVID. The median age at onset of symptoms was 24 (0–66), 33 (4–70) at diagnosis, and 37 (18–73) years at the time of analysis. The median diagnostic delay for the entire study population was 6 (0–57) years. However, this delay was higher in patients with symptom onset before the year 2000 than after the year 1999 [15 (0–57) vs. 3 (0–19) years; *p* < 0.001]. Comparing patients (median ≤ 6 years, *N* = 53) with short diagnostic delay (SDD) and those (median > 6 years, *N* = 50) with long diagnostic delay (LDD), the LDD group had a statistically significant higher incidence of infections of the lower respiratory tract before diagnosis (90.0 vs. 71.70%). During the entire observation period, cytopenias (44.00 vs. 22.64%), granulomatous lesions (28.00 vs. 11.32%), and solid tumors (14.00 vs. 1.89%) were significantly more frequent in the LDD group. In conclusion, we found a significant reduction in the median diagnostic delay in Polish CVID patients with disease onset in the last two decades.

## Introduction

Primary immunodeficiencies (PIDs) are rare diseases. Because of their innate nature, they are diagnosed mainly in childhood (1). More than half of PID cases are associated with a defect in antibody production or function (2). In this group, the most common symptomatic deficiency is common variable immunodeficiency (CVID) (1, 3). CVID is a heterogeneous group of disorders characterized by recurrent upper and lower respiratory tract infections, which occur in more than 85% of patients (4). Besides, up to 70% of patients have at least one non-infectious manifestation, such as autoimmunization, granulomatous lesions, unexplained polyclonal lymphoproliferation, enteropathy, or malignancy (5–7).

Epidemiological data indicate that CVID has two peaks of onset. The first peak occurs in childhood, and the second peak occurs in the third or fourth decade of life. However, symptoms of CVID can start at any time of life, even in elderly patients (8). In Europe, 60% of CVID diagnosis occurs in adults (Table 1).

**Table 1:** Summary of most relevant CVID epidemiological studies in selected countries.

**Country**	**Reported period (years)**	**Number of patients**	**Age at time of analysis**	**Age at** **onset**	**Age at** **diagnosis**	**Diagnostic delay**	**% of patients diagnosed as adults**	**References**
Denmark	1994–2013	179	50.1± 17.0	29 (IQR; 3–87)	40 (IQR; 29–56) min 4; max 87	7 (IQR; 3–17)	–	Westh et al. (9)
Germany	2012–2017	728	40 (3–88)	–	Max 79	Mean: 7.35 median: 3	65%	El-Helou et al. (1)
Italy	1985–2015	75	50.08 ± 15.81; Median: 49	32 [17.82]^*^	40 [16.01]^*^	7 (IQR; 3–13)	–	Graziano et al. (10)
Poland	2017	77	39.19 ± 13.61	22.16 ± 14.32	32.29 ± 14.94	10.13 ± 10.53	76.6%	Wiesik-Szewczyk et al. (11)
Switzerland	2008–2014	98	–	–	–	Median: 5.95	87.5%	Marschall et al. (12)
United Kingdom	(2008^**^) 2012–2017	1,404	–	–	–	4 (IQR; 1–10) 4 (0–69)	–	Shillitoe et al. (3)
Europe (23 countries)	2004–2014	2,700	–	18 (0–81) 22.4 ± 19.0	31 (4–89)	4 (0–69) 8.8 ± 11.4	69.5%	Odnoletkova et al. (13)
Europe (16 countries)	2004–2012	2,212	–	–	–	4.1 (IQR; 1–11.8)	86.7%	Gathmann et al. (8)

If not otherwise indicated, data are presented as median (minimum-maximum) or median (interquartile range—IQR) or mean ± SD.

* Median [SD].

** *United Kingdom Primary Immunodeficiency (UKPID) registry exists from 2008*.

Due to low awareness among physicians regarding PID in adults, the onset of symptoms in adulthood, and a heterogeneous clinical picture of CVID, it may take up to several years to establish a proper diagnosis (14). The analysis of nearly 3,000 CVID cases showed a relationship between diagnosis delay and a higher risk of death [1.04 (1.02, 1.06), *p* = 0.0003], and organ complications (13). Aghamohammadi et al. demonstrated that the delay in diagnosis correlated significantly with the severity of the infection and the number of hospitalizations in children with primary antibody deficiencies, including CVID (15). Diagnostic delay of CVID generates high socioeconomic costs. According to Sadeghi et al., a diagnosis of CVID in a single patient can save US$ 6500 annually (16).

Similar to other rare diseases, data on CVID epidemiology are derived mainly from registries. In the last decade, several papers have been published, analyzing data from the ESID register (8, 13) or national registers (1, 3, 9, 10, 12). According to these studies, the diagnostic delay ranges between 3 and 9 years (Table 1). The period between the onset of first symptoms and CVID diagnosis is reportedly significantly shortened after 2000 in Spain (8) and the United Kingdom (3). In several other countries, there has been a tendency to shorten the delay of diagnosis, but the differences have not reached statistical significance (1, 8).

In Poland, we have very limited knowledge regarding CVID epidemiology. Considering the estimated prevalence of 1:25,000–1:50,000 and the population of Poland, which is about 38.386 million (17), there should be about 760–1,500 patients with CVID in this country. According to available data, 78 new cases were identified in 2014 (including 49 in children, 29 in adults) (18), and the median diagnostic delay in one of the pediatric centers (Kraków, 32 patients) was 1.8 years (8). According to data published in 2018, in a group of 77 adult Polish CVID patients, the mean diagnosis delay was 10.13 ± 10.53 years (19).

This study aimed to determine the length of the diagnostic delay of CVID in a group of Polish adult patients and compare groups of patients with short (SDD) and long diagnostic delay (LDD).

## Materials and Methods

### Study Population

Data of CVID patients were collected from May 24, 2017, to December 31, 2019, using an internet database. The database did not contain personal data, and the patients were identified by code numbers. Only the attending physician of a particular patient could link the code number and patient's data. Entries older than 12 months were updated every year.

The study group consisted of patients treated under the Polish Ministry of Health's drug programs B.62 and B.78. A drug program is defined as follows: “guaranteed compensation, including therapies with innovative, expensive active substances, which are not financed by other guaranteed benefits. The treatment is carried out in selected disease entities and includes a strictly defined group of patients” (20). Within the aforementioned drug programs, immunoglobulin replacement therapy and monitoring are reimbursed for patients with primary humoral immunodeficiencies. Patients were treated at four immunological centers specializing in the care of adult patients with primary immunodeficiencies (Department of Allergology, Clinical Immunology and Internal Diseases, Ludwik Rydygier Collegium Medicum in Bydgoszcz Nicolaus Copernicus University in Torun, Bydgoszcz; Department of Internal Medicine, Connective Tissue Diseases and Geriatrics, Medical University of Gdansk, Gdansk; Outpatient Clinic for the Immunological and Hypercoagulable Diseases, The University Hospital in Krakow, Cracow; and Department of Internal Medicine, Pneumonology, Allergology and Clinical Immunology, Central Clinical Hospital of the Ministry of National Defense, Military Institute of Medicine, Warsaw). All patients met the Registry Working Definitions of the European Society for Immunodeficiencies (ESID) for CVID (21).

Of note, the most important epidemiological and clinical data are available as a Data Sheet, in the Supplementary Materials.

### Data Collection

We collected data on the age of onset of the first symptoms, age at the time of CVID diagnosis, immunoglobulin (Ig) levels at the time of diagnosis, and type of infections before diagnosis. We also recorded the most important organ complications and co-morbidities associated with CVID from the time of the first symptoms until the data were entered in the database or updated. The year in which the first symptoms occurred was considered as the year in which the frequency of infection increased, a severe infection requiring hospitalization or intravenous antibiotic treatment, or the year in which symptoms of autoimmunity, polyclonal lymphoproliferation, or malignancy occurred. The age of onset of the first symptoms and that at the time of diagnosis was calculated as the difference in years between the year of birth of the patient and the year in which the event occurred. The diagnostic delay was calculated as the difference of full years between the years of onset of symptoms and diagnosis.

Due to the median delay in diagnosis for all patients (6 years), the cohort was divided into the following groups: SDD (median delay ≤ 6 years; *N* = 53) and LDD (median delay > 6 years; *N* = 50). The groups were compared in terms of age of first symptoms, age of diagnosis, as well as IgG, IgA, and IgM levels at the time of diagnosis, the incidence of infection in the period before diagnosis, and incidence of complications and co-morbidities throughout the observation period.

### Statistical Analysis

The normality of the observed values was tested using the Shapiro-Wilk test. For the continuous variables, mean and standard deviation were calculated if they followed a normal distribution; for non-normal distributions, the median (minimum to maximum) was used. Continuous variables were analyzed using Student's T, Mann-Whitney-U, and Kruskal–Wallis tests. Categorical variables were analyzed using the Chi-square test. For all data analyses, differences were considered statistically significant when *p* < 0.05. The statistical analysis was performed using the STATISTICA software (TIBCO Software Inc. Palo Alto, CA, USA), version 13.

### Ethics Statement

The study was approved by the Ethics Committee of the Military Institute of Medicine, Warsaw (7/WIM/2020). All patients provided written consent to the collection and analysis of their demographic and medical data.

## Results

### Characterization of CVID Patient Population

This study consisted of 103 adult patients, including 46 women (44.7%) and 57 men (55.3%) with CVID. At the time of data analysis, their median age was 37 (18–73) years.

**Figure 1:**
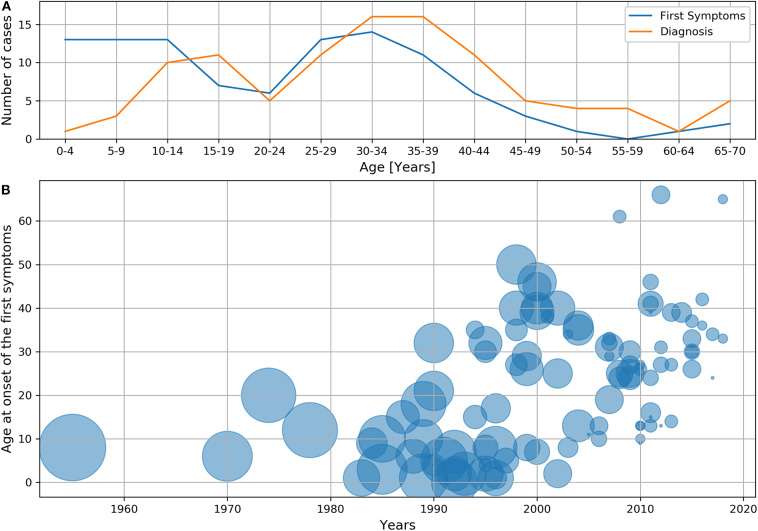
Age of first symptoms or diagnosis and diagnostic delay: **(A)** Age of first symptoms and age of diagnosis. **(B)** Diagnostic delay depending on the age and year at which the first symptoms occurred. The diameter of the circle corresponds to the delay expressed in years, and the center indicates the age and year at which the first symptoms occurred.

The first symptoms of the disease commonly appeared from 0 to 14 years (39 patients; 37.9%) and 25–39 years of age (38 patients; 36.9%). Additionally, for the age of diagnosis, a bimodal distribution was observed. CVID was diagnosed in the highest percentage of patients at 10–19 years (21 patients; 20.4%) and 30–39 years of age (32 patients; 31.1%) (Figure 1A).

The median age at onset of symptoms was 24 (0–66) years. The first symptoms occurred in 44 patients (42.7%) before 18 years of age. In 41 patients (39.8%), disease onset occurred before the year 2000. In the decades following 1980, the mean age of patients at the onset of symptoms increased from 8.11 ± 5.97 to 29.7 ± 14.1 years (Table 2).

**Table 2:** Mean delay of CVID diagnosis and mean age of patients in subsequent decades, depending on the age of first symptoms and the age of diagnosis.

**Years**	**First symptoms**			**Diagnosis**		
	**N**	**Diagnostic delay**	**Age**	**N**	**Diagnostic delay**	**Age**
1950–1979	4 (3.88%)	41.5 ± 10.8	11.5 ± 6.19	–	–	–
1980–1989	9 (8.74%)	21.4 ± 7.58	8.11 ± 5.97	–	–	–
1990–1999	28 (27.18%)	13.1 ± 7.85	16.1 ± 14.7	5 (4.85%)	4.80 ± 4.60	16.0 ± 14.4
2000–2009	29 (28.16%)	7.66 ± 4.97	27.7 ±1 3.5	24 (23.30%)	8.96 ± 7.29	25.1 ± 11.2
2010–2019	33 (32.04%)	2.24 ± 1.77	29.7 ± 14.1	74 (71.84%)	10.6 ± 11.3	36.3 ± 14.9
*p* value^*^	–	<0.001	<0.001	–	0.535	<0.001

The values are presented as number (%) or mean ± SD.

* *Kruskal-Wallis test*.

The median age at the time of CVID diagnosis was 33 years (4–70). In subsequent decades, the diagnosis was established in increasingly older patients. The age of diagnosis at specified intervals is presented in Table 2. Childhood (<18 years of age) diagnosis was established in 23 patients (22.3%). In 74 patients (71.8%), CVID was diagnosed between 2010 and 2019 (Table 2). In 7 cases (6.8%), including five patients under 18 years of age, the diagnosis was established in the same calendar year in which the first symptoms occurred.

The median diagnostic delay of the study group was 6 (0–57) years (mean 9.91 ± 10.3 years). In men, the median delay was 9.0 (0–39) years and 5.0 (0–57) years in women. These differences were not statistically significant (*p* = 0.191). The mean diagnostic delay was 41.5 ± 10.8 years in patients whose first symptoms occurred between 1950 and 1979. In subsequent decades, this delay systematically decreased, and from 2010 to 2019, it was 2.24 ± 1.77 years (Table 2). The median delay in patients with first symptoms before 2000 was 15 (0–57) years and 3 (0–19) years after 1999 (*p* < 0.001). Further, we observed a reduction in diagnostic delay, even if the first symptoms occurred in elderly patients (Figure 1B), which was the most prominent after the year 2000.

In the decades following 1990, the mean delay assessed at the time of diagnosis increased from 4.80 ± 4.60 to 10.6 ± 11.3 years. The differences in subsequent analyzed periods were not statistically significant (Table 2).

### Comparison of Patients With SDD and LDD

There were statistically significant differences between groups of patients with SDD and LDD (Table 3) in the age of appearance of first symptoms [27.0 (1–66) vs. 15.0 (0–50) *p* = 0.004], the age at which the diagnosis was established [31.0 (4–70) vs. 34.5 (12–70) *p* = 0.04], and the age at the time of analysis [35.0 (18–73) vs. 41.0 (19–72) *p* = 0.036]. In both groups, at the time of diagnosis, IgG, IgA, and IgM levels were comparable. Infections of the lower respiratory tract occurred in a significantly higher percentage of patients in the LDD group than in the SDD group (90.0 vs. 71.70%; *p* = 0.019). There were no significant differences in other infections between SSD and LDD groups.

**Table 3:** Comparison of patients with short and long diagnostic delay.

	**Short Diagnostic Delay Median ≤ 6 years**	**Long Diagnostic Delay Median > 6 years**	***p***
N (%)	53 (51.46%)	50 (48.54%)	–
Women: men N (%)	28:25 (52.8%:47.2%)	18:32 (36.0%:64.0%)	0.086
Age at the first symptoms [years]	27.0 (1–66)	15.0 (0–50)	**0.004**
Age at the time of diagnosis [years]	31.0 (4–70)	34.5 (12–70)	**0.040**
Age at the time of analysis [years]	35.0 (18–73)	41.0 (19–72)	**0.036**
IgG at the time of diagnosis [mg/dl]	138.0 (0–543)	204.0 (0–640)	0.870
IgM at the time of diagnosis [mg/dl]	15.0 (0–93)	10.5 (0–903)	0.638
IgA at the time of diagnosis [mg/dl]	5.0 (0–67.5)	6.0 (0–53)	0.509
**INFECTIONS BEFORE CVID DIAGNOSIS** ***N*** **(%)**			
Upper respiratory tract (except sinusitis)	50 (94.34%)	49 (98.0%)	0.336
Nose and paranasal sinuses	48 (90.57%)	47 (94.0%)	0.515
Lower respiratory tract	38 (71.70%)	45 (90.0%)	**0.019**
Middle ear	40 (75.47%)	35 (70.0%)	0.532
Gastrointestinal tract	12 (22.64%)	11 (22.00%)	0.938
Urinary tract	7 (13.21%)	8 (16.00%)	0.688
Skin and subcutaneous tissue	9 (17.00%)	5 (10.00%)	0.301
Generalized infection/sepsis	6 (11.00%)	11 (22.00%)	0.154
**CVID COMPLICATIONS AND CO-MORBIDITIES** ***N*** **(%)**			
Any autoimmunization	22 (41.51%)	29 (58.00%)	0.094
Cytopenia	12 (22.64%)	22 (44.00%)	**0.021**
Thrombocytopenia	9 (16.98%)	13 (26.00%)	0.264
Enteropathy	7 (13.2%)	3 (6.0%)	0.217
Bronchiectasis	13 (25.49%)	8 (16.00%)	0.240
Polyclonal lymphoproliferation (Lymphadenopathy, GLILD, etc.)	16 (30.19%)	18 (36.00%)	0.531
Granulomatous lesions	6 (11.32%)	14 (28.00%)	**0.032**
Splenomegaly	4 (7.55%)	8 (16.00%)	0.181
Malignancy total	3 (5.66%)	9 (18.00%)	**0.049**
Lymphoma	2 (3,77%)	2 (4.00%)	0.953
Solid tumors	1 (1.89%)	7 (14.00%)	**0.022**

*Bolded p-values indicate statistical significance (p < 0.05)*.

Cytopenias (44.00 vs. 22.64% *p* = 0.021), granulomatous lesions (28.00 vs. 11.32% *p* = 0.032), and malignancies (18.00 vs. 5.66% *p* = 0.049), including solid tumors (14.0 vs. 1.89% *p* = 0.022), were significantly more frequent in the LDD group than in the SDD group (Table 3). Autoimmunization, thrombocytopenia (as the most frequent cytopenia), polyclonal lymphoproliferation, splenomegaly, and lymphoma were more frequent in the LDD group than in the SDD group, although these differences were not statistically significant. Further, in the SDD group, bronchiectasis, and enteropathy were more frequent. However, these differences were not significant.

## Discussion

According to data published after 2010, the median delay in CVID diagnosis (Table 1) ranges from 3 years in Germany (1) to 7 years in Italy (10) and Denmark (9). In the 23 European countries analyzed together, the median diagnostic delay is 4 (0–69) years, and the mean is 8.8 ± 11.4 years (13).

In our group, the median delay was 6 years. However, for patients whose first symptoms appeared between 2010 and 2019, the mean delay was shortened to slightly over 2 years. According to the 2014 ESID registry, a significant shortening of the median delay was achieved only in Spain (9.0 vs. 4.6 years) (8). Furthermore, the United Kingdom demonstrated a statistically significant but weak correlation for a decrease in diagnostic delay over time from 2012 to 2017 (3).

At present, in Poland, CVID diagnosis is more rapid than that before 2000, even in elderly patients. However, compared to other European countries, a lower percentage of patients whose diagnosis was established in the year in which the first symptoms occurred remains (6.8 vs. 16.0%) (13).

In the group of Polish patients, we observed an increase in the mean diagnostic delay, assessed at the time of diagnosis, over the last three decades (Table 2). This finding may result from patients who had undiagnosed CVID symptoms for several years. A similar phenomenon occurred in Europe, in which the mean delay assessed at the time of diagnosis before and in 1980 was 7.4 years, and in and after 2000 was 8.8 years (13).

Most CVID cases were identified in Poland after 1999, which is higher than in other European countries (95.15 vs. 69.1%) (13). This striking difference was possibly due to efforts by the Polish Ministry of Health, which provided reimbursements of immunoglobulin treatment for patients with primary immunodeficiency in 2015 as part of its drug programs (11). Additionally, after year 2000 new Polish centers for adult PID patients were established that improved accessibility for clinical immunologist consultations.

Comparing the group of patients with SDD and LDD, statistically significant differences were found between the age of first symptoms and the age at which CVID was diagnosed. Patients from the SDD group were older at the time of onset, while they were younger at the time of diagnosis compared to patients from the LDD group. This could be due to at least three reasons. First, at the time of analysis, there were no patients under 18 years old in the study group, which increased the median age at the onset of the first symptoms and decreased the median age at which the diagnosis was established. Second, in patients who were older at the time of data analysis, the first symptoms could have occurred in childhood, which lowered the age median when the first symptoms occurred in the LDD group. Third, before 2000, the delay in diagnosis was considerably longer than in recent years, which resulted in patients waiting longer to be diagnosed, even if symptoms occurred at a young age.

A statistically significant difference in the percentage of patients who had infections before CVID diagnosis was found exclusively in the case of lower respiratory tract infection, which was higher in the LDD group. This finding could be due to the delay in the initiation of IgG substitution.

Many studies have highlighted the occurrence of numerous complications and co-morbidities during the course of CVID (6, 8, 13, 22, 23). In this study cytopenias, granulomatous lesions, solid tumors, and neoplastic diseases were more frequent in the LDD group. The more frequent occurrence of the above-mentioned non-infectious complications in the LDD group could be a consequence of several phenomena. Due to low awareness among physicians of PID in adults, in patients who do not present with recurrent infections, diagnostics focus may divert from CVID. For instance, granulomatous lesions may occasionally be misdiagnosed as sarcoidosis (24). Moreover, the analysis of 21 patients with CVID and idiopathic thrombocytopenic purpura showed that only 19% of patients were diagnosed with immunodeficiency before the diagnosis of ITP (25). The more frequent occurrence of cancers, in the LDD group may be associated with the patients' older age and longer disease duration. In addition, Kiaee et al. performed a meta-analysis showing that CVID patients diagnosed with malignancy were older at the time of diagnosis, relative to patients without malignancy (5).

Recurrent lung infections are a recognized risk factor for bronchiectasis (26). Although the percentage of patients with lower respiratory tract infections was higher in the LDD group examined here, the percentage of patients with bronchiectasis in the LDD group was lower than that in the SDD group. This discrepancy may have resulted from the lack of discrimination between patients affected by chronic conditions from those with sporadic lower respiratory tract infections. Nevertheless, other cofactors, such as very low IgA or IgM level, or low neonatal Fc receptor expression, reportedly contribute to bronchiectasis (26). It can be assumed that the occurrence of bronchiectasis may have directed and accelerated the diagnosis toward CVID.

In the available literature, data on the relationship between delayed diagnosis and the occurrence of individual complications are unclear. Odnoletkova et al. reported in a group of 2700 patients with CVID that the diagnostic delay is associated with a higher risk of death, bronchiectasis, solid tumors, and enteropathy (13). In contrast, Razi et al., in a meta-analysis of 8,535 patients, did not show a correlation between delayed diagnosis and the occurrence of bronchiectasis. The incidence of bronchiectasis in the group of patients with 3 years or longer delay compared to the group with a shorter delay did not show a statistical difference (37.4 vs. 25.8%) (23). Further, in a group of 40 patients, researchers found a correlation between diagnosis delay and bronchiectasis (*r* = 0.323, *p* = 0.042), but did not confirm the correlation between chronic diarrhea and diagnostic delay (27).

Undoubtedly, further research on the relationship between the delayed diagnosis and occurrence of complications is necessary for a larger patient population.

Our study had a few limitations. Only adult patients were included in the analysis, which may have resulted in the overestimation of some indicators, such as the age of first symptoms or that of diagnosis. Further, four clinical, immunological centers participated in the study. Therefore, the analysis consisted of only a segment of Polish patients with CVID. Additionally, the relatively small number of patients made it difficult to analyze the incidence of rare complications statistically. Finally, for patients whose first symptoms occurred a long time ago (even in the 1950s), we had incomplete medical documentation, especially from the initial period of the disease. In a few cases, the type of infection before establishing a diagnosis was based on the oral records of the patients.

## Conclusion

To the best of our knowledge, this is the first study on the delay in CVID diagnosis in the largest group of Polish patients. Notably, in recent years, the median time of delay in CVID diagnosis in Poland has significantly shortened and reached values comparable to that of other European countries. Presently, even an adult patient whose first symptoms occur at a late age can be diagnosed more quickly. However, further efforts are needed to assess the epidemiological and clinical landscape of patients with CVID and other primary immunodeficiencies (PID). To this end, we plan to establish the Polish Register of Primary Immunodeficiency Deficiencies in Adults (POLPIDA) that will facilitate a better, more comprehensive understanding of the needs of Polish patients for the diagnosis and therapy of PID and especially CVID. We believe that our continued effort will help reduce the incidence and severity of clinical complications.

## Data Availability Statement

All datasets generated for this study are included in the article/Supplementary Material.

## Ethics Statement

The studies involving human participants were reviewed and approved by Ethics Committee of the Military Institute of Medicine, Warsaw, Poland. The patients/participants provided their written informed consent to participate in this study.

## Author Contributions

MZ and EW-S designed the study and wrote the first draft of the manuscript. This text was produced with an equal contribution of both authors. MZ, EW-S, AM-B, and KN-B collected data and performed managed the literature searches. MZ performed the statistical analyzes. AM-B, KN-B, ZZ, and KJ-R performed a critical revision of the manuscript for intellectual content. All authors have read and agreed to the published version of the manuscript.

## Conflict of Interest

The authors declare that the research was conducted in the absence of any commercial or financial relationships that could be construed as a potential conflict of interest.
